# Visualizing phosphatidic acid and diacylglycerol at the nuclear envelope in fission yeast

**DOI:** 10.1007/978-1-0716-4714-1_7

**Published:** 2025-01-01

**Authors:** Sherman Foo, Snezhana Oliferenko

**Affiliations:** 1 Randall Centre for Cell and Molecular Biophysics, School of Basic and Medical Biosciences, King’s College London, London SE1 1UL, UK; 2 The Francis Crick Institute, 1 Midland Road, London, NW1 1AT, UK

**Keywords:** Phosphatidic Acid, Diacylglycerol, Nuclear Envelope, Fission Yeast, Microscopy

## Abstract

Although the outer membrane of the nuclear envelope is continuous with the endoplasmic reticulum, temporally regulated and functionally significant differences in membrane lipid composition may exist between the two nuclear membranes and between the outer nuclear membrane and the endoplasmic reticulum. Biochemical approaches to probing lipid composition are challenged when lipid dynamics must be analyzed with fine spatiotemporal resolution, for instance within the cell cycle of a single cell. Here we describe a method to probe the distribution of phosphatidic acid and diacylglycerol, two interconvertible biosynthetic precursors for other membrane glycerophospholipids, in living cells of the model fission yeast *S. pombe*. We show how genetically-encoded fluorescent biosensors can be constructed and optimized, and present a protocol to probe and quantify phosphatidic acid and diacylglycerol levels specifically at the inner nuclear membrane.

## 1 Introduction

Lipids are a major class of biomolecules present in all organisms. They form the bulk of biological membranes delimiting cells and eukaryotic organelles, determine the function of membrane-associated proteins, and may act as signaling and storage molecules. Lipid repertoire differs between, and even within, different cellular membranes, which is likely achieved through a combination of transport and compartmentalized metabolism [1]. Although the nuclear envelope (NE) can be considered a subdomain of the endoplasmic reticulum (ER), its two constituent membranes may differ in metabolic capabilities and the abundance of specific lipids. For instance, the inner nuclear membrane (INM), traditionally thought to be metabolically silent, has been recently shown to house lipid biosynthetic reactions, such as the synthesis of storage lipid triacylglycerol [2], and the formation of CDP-choline, the rate-limiting step in the production of phosphatidylcholine [3].

Dynamic changes in lipid distribution across different membranes may have profound implications for cellular physiology [4–10], but they are notoriously difficult to study [1, 11]. Recent exciting mass-spectrometry-based methodologies and the advent of fluorescent and photoactivatable lipid derivatives have begun to tackle the complexity of lipid transport and metabolism in living cells [12–16]. Yet, these strategies are technically demanding and/or struggle with reporting changes in the subcellular distribution of endogenous lipids in single cells on functionally relevant timescales. A simple alternative is utilizing genetically encoded fluorescent lipid biosensors, typically built on protein domains recognizing specific lipid classes. Such biosensors, e.g., for phosphatidic acid (PA), diacylglycerol (DG), phosphatidylcholine and sterols, have been used in a range of experimental systems [4, 17–26].

We have developed a suite of nuclear and cytoplasmic PA and DG biosensors for the use in the fission yeasts *Schizosaccharomyces pombe* and *Schizosaccharomyces japonicus* [4]. This advance has allowed us to discover the critical roles of the DG kinase Dgk1 and the DG cholinephosphotransferase Ept1 in boosting glycerophospholipid synthesis, which enables NE expansion required for “closed” mitosis of *S. pombe*. Importantly, we have optimized biosensor designs used previously in other experimental systems, and provided suggestions on their expression levels [4].

**Figure 1:**
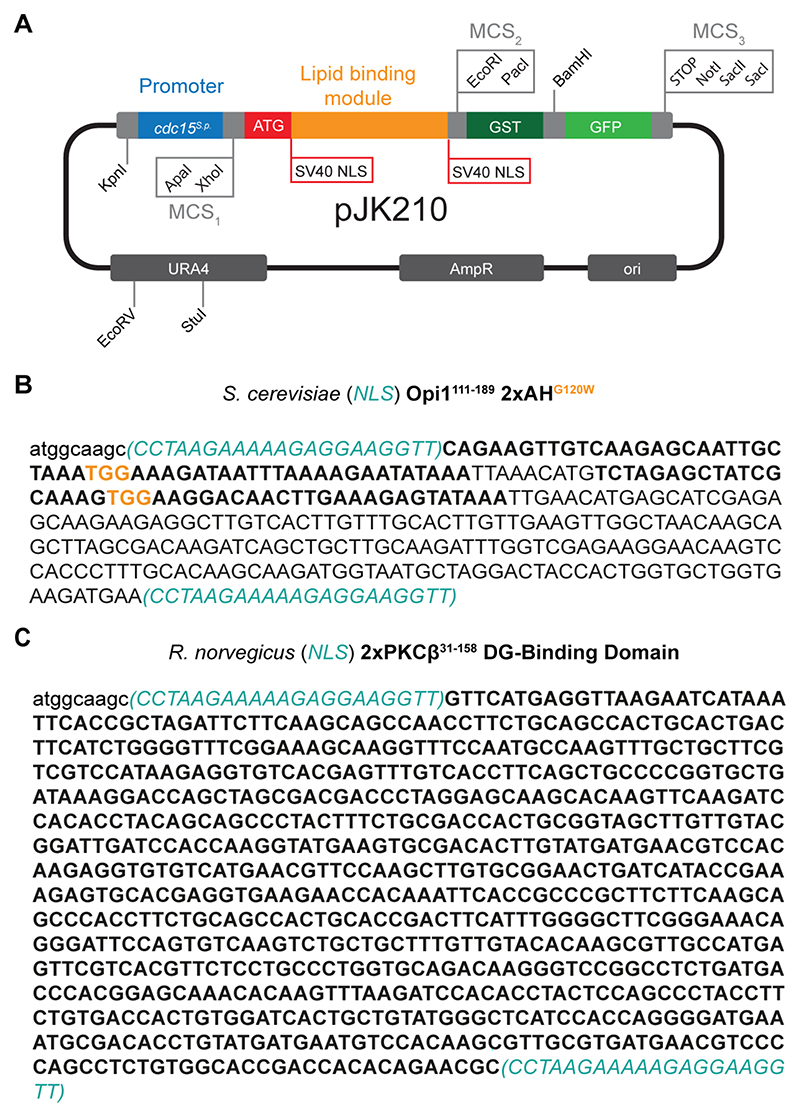
(A) Diagram depicting the plasmid design for expressing lipid sensors in the study [4]. The lipid sensors consist of a lipid-binding module (reengineered duplicated *S. cerevisiae* Opi1 PA-binding amphipathic helix or duplicated *R. norvegicus* PKCβ DG-binding domain) under the control of the *S. pombe cdc15* promoter, with a C-terminal GST tag and GFP. Restriction enzyme sites and the multiple cloning sites (MCS) are depicted in grey. In the NLS versions of the sensors, the lipid-binding module is flanked by two SV40 NLS motifs on both ends. (B) DNA sequence of the PA lipid sensor module. It consists of two consecutive *S. cerevisiae* Opi1 amphipathic helices each containing a G120W point mutation. The amphipathic helices are indicated in bold, mutations indicated in orange, and SV40 NLS motifs in cyan. (C) DNA sequence of the DG lipid sensor module. The DG lipid sensor consists of two consecutive *R. norvegicus* PKCβ DG-binding domains indicated in bold and the SV40 NLS motifs in cyan.

**Figure 2:**
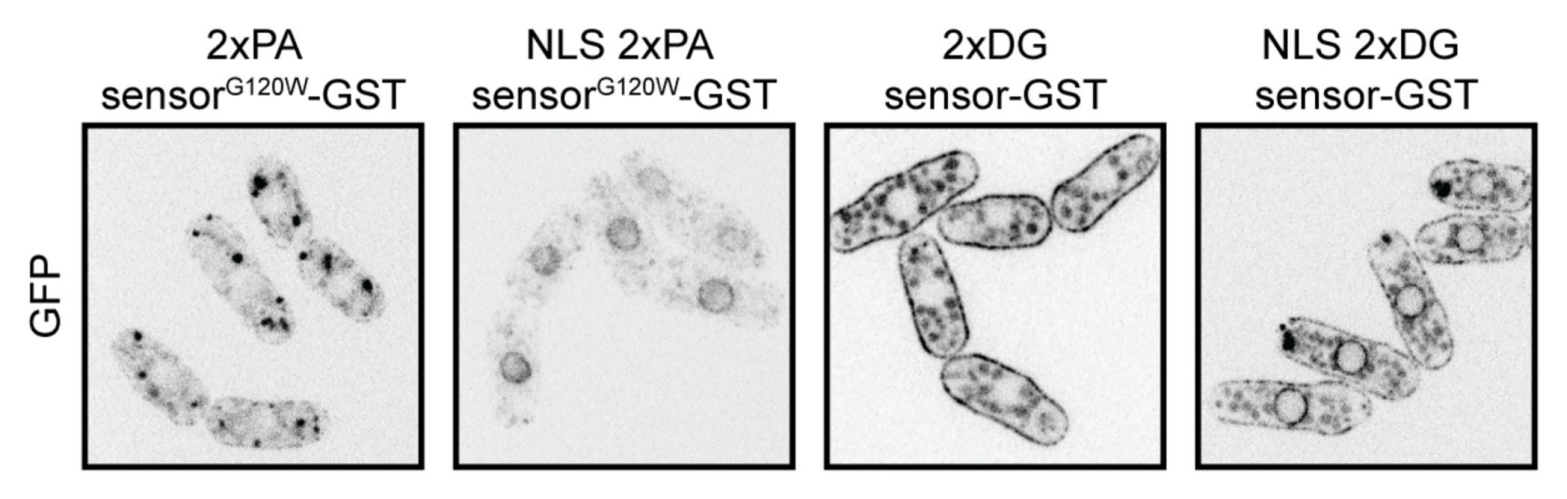
Single-plane spinning-disk confocal images of *S. pombe* expressing the cytoplasmic and NLS PA and DG GFP-based sensors. Scale bars: 5 µm.

The PA sensor was constructed by removing an endogenous nuclear localization sequence (NLS) within the codon-optimized Q2 helix of the *S. cerevisiae* transcriptional factor Opi1 [24], introducing a G120W point mutation predicted to increase PA-binding selectivity [23], and duplicating the helix to stabilize it at the membrane. The DG sensor was based in duplicated codon-optimized rat protein kinase C DG-binding domain [22]. In our published designs, both biosensors were expressed under the control of the medium-strength *S. pombe cdc15* promoter and tagged at the C-terminus with a Glutathione S-Transferase (GST) tag and Green Fluorescent Protein (GFP). GST has served as a means of increasing protein bulk, preventing passive protein diffusion through the nuclear pores. Appending or omitting the SV40 NLS at both ends of the lipid-binding module allowed us to probe lipid enrichment at the INM or in the cytoplasm, respectively. Both sensor designs have been validated by modulating the cellular abundance of PA and DG, and by mutating amino acids known to mediate binding to the target lipids [4] (Figure 1-2).

Here, we describe how basic manipulation of the biosensors can be performed, followed by the protocol to probe PA and DG enrichment at the INM in *S. pombe*. We will replace the GFP fluorophore with mCherry as an example workflow. Similarly, the promoter, GST tag, or selection marker can be swapped out using appropriate DNA fragments and restriction enzymes as necessary. In terms of choosing endogenous promoters of different strengths, PomBase, an excellent curated database, contains the regulatory context of all *S. pombe* genes [27], and there is increasing amount of information on *S. japonicus*, in a sister database JaponicusDB [28].

## 2 Materials

### 2.1 Polymerase Chain Reaction

1)   Template DNA (e.g., mCherry-containing plasmid)
2)   Forward and reverse primers (100μM)
3)   KAPA HiFi PCR Kit (Roche)
4)   dNTP mix (10mM)
5)   PCR-grade water
6)   8-strip PCR tubes and caps
7)   PCR machine
8)   UltraPure Agarose (Thermo Scientific)
9)   Tris Acetate EDTA (TAE) buffer (Thermo Scientific)
10) GelRed Nucleic Acid Stain (Sigma-Aldrich)
11) Sub-Cell GT DNA electrophoresis cell and combs (Bio-Rad)
12) PowerPac Basic Power Supply (Bio-Rad)
13) DNA ladder
14) GelDoc system
15) Macherey-Nagel NucleoSpin Gel and PCR Clean-up Kit (Fisher Scientific)
16) Ethanol
17) Benchtop centrifuge for 1.5mL microcentrifuge tubes
18) Nanodrop (Thermo Scientific)
19) Microcentrifuge tubes
20) -20°C freezer

### 2.2 Molecular Cloning

1)   PCR insert (1μg)
2)   Vector (1μg)
3)   Restriction enzymes and buffer (NEB)
4)   PCR-grade Water
5)   Microcentrifuge tubes
6)   Heat block
7)   UltraPure Agarose (Thermo Scientific)
8)   Tris Acetate EDTA (TAE) buffer (Thermo Scientific)
9)   GelRed Nucleic Acid Stain (Sigma-Aldrich)
10) Sub-Cell GT DNA electrophoresis cell and combs(Bio-Rad)
11) PowerPac Basic Power Supply (Bio-Rad)
12) DNA ladder
13) GelDoc system
14) Scalpel
15) Macherey-Nagel NucleoSpin Gel and PCR Clean-up Kit (Fisher Scientific)
16) Ethanol
17) Benchtop centrifuge for 1.5mL microcentrifuge tubes
18) Nanodrop (Thermo Scientific)
19) -20°C freezer
20) T4 DNA ligase and buffer (NEB)
21) 5-alpha Competent *E. coli* cells (NEB)
22) SOC media
23) LB plates (100μg/mL ampicillin)
24) 37°C incubator

### 2.3 Miniprep and Analytical Digestion

1)   13mL snap-cap tubes
2)   LB media (100μg/mL ampicillin)
3)   37°C shaking incubator
4)   Benchtop centrifuge for 15mL tubes
5)   ThermoScientific GeneJet Miniprep Kit
6)   Ethanol
7)   Restriction enzymes and buffer (NEB)
8)   Microcentrifuge tubes
9)   Heat block
10) 6x gel loading dye (NEB)
11) UltraPure Agarose (Thermo Scientific)
12) Tris Acetate EDTA (TAE) buffer (Thermo Scientific)
13) GelRed Nucleic Acid Stain (Sigma-Aldrich)
14) Sub-Cell GT DNA electrophoresis cell and combs (Bio-Rad)
15) PowerPac Basic Power Supply (Bio-Rad)
16) DNA ladder
17) GelDoc system
18) -20°C freezer

### 2.4 Yeast molecular genetics and husbandry

1)   Glass flasks for yeast cultures
2)   Benchtop centrifuge for 50mL tubes
3)   Benchtop centrifuge for 1.5mL tubes
4)   Benchtop spectrometer
5)   Reagent grade water
6)   Microcentrifuge tubes
7)   LiAc-TE buffer (0.1M lithium acetate, 10mM Tris pH 7.5, 1 mM EDTA)
8)   LiAc-TE-PEG buffer (LiAc-TE, 40% PEG4000)
9)   Carrier DNA (10mg/mL Sonicated Salmon Sperm DNA)
10) Heat block
11) DMSO
12) Edinburgh Minimal Medium (EMM) plates supplemented with leucine, histidine and adenine (225mg/L each)
13) 30°C incubator
14) MasterPure Yeast DNA Purification Kit (LGC group)
15) Ethanol
16) Isopropanol
17) Forward and reverse genotyping primers (100μM)
18) PCR materials (*see* Subheading 2.1)
19) Cryotubes
20) Glycerol
21) -80°C freezer

### 2.5 Imaging

1)   Glass microscopy slides
2)   Coverslips
3)   Fluorescence microscope system, e.g., spinning disk confocal
4)   UltraPure agarose
5)   Edinburgh Minimal Medium (EMM) medium supplemented with leucine, histidine and adenine (225mg/L each)
6)   Yeast Extract with Supplements (YES) medium
7)   Heat block
8)   Microcentrifuge tubes
9)   Benchtop centrifuge
10) Wax or nail polish

## 3 Methods

### 3.1 Background information

1) The following lipid biosensor constructs and *S. pombe* strains expressing them have been extensively validated [4]:
2) Figure 1A shows an overview of the design and restriction sites used in the construct of the sensors. The DNA sequences of the PA and DG lipid sensors can be found in Figures 1B and 1C respectively. All sensors are cloned into pJK210-based plasmid backbone for integration into the *ura4* locus of *S. pombe* [29].
3) Representative spinning disk confocal microscopy images of the strains in Table 2 can be found in Figure 2.
4) The presence of restriction sites flanking the promoter, lipid binding module, GST biochemical tag and GFP fluorophore allows for the replacement of any of the components with suitable alternatives (e.g., different promoter, alternative biochemical tags such as MBP, HA; alternative fluorophores such as mCherry or mNeonGreen) (*see* Notes 1 and 2). In the following sections we will discuss swapping the GFP fluorophore to mCherry as an example molecular cloning workflow. Alternatively, Gibson assembly or similar approaches can be used to swap out these components as necessary [30].
5) Plasmids and yeast strains can be obtained via request from the authors.

### 3.2 PCR of region of interest

1) Design primers for PCR of a fluorophore of interest. For further details on good primer design, refer to this in-depth guide by Álvarez-Fernández [31] or Dieffenbach and colleagues [32]. In this section, we use mCherry as an example workflow, to enable co-localization of a protein of interest tagged with GFP together with an mCherry-tagged lipid sensor.
2) Primers targeting the 5’ and 3’ ends of the mCherry gene are designed and procured commercially. The forward primer should include a BamHI restriction site at its 5’ end while the reverse primer contains a Stop codon, followed by a NotI (or SacII or SacI) restriction site at its 3’ end. Check that the sequence of the fluorophore of interest does not contain these restriction sites.
3) Using appropriate mCherry-containing plasmid template, perform PCR reaction according to the manufacturer’s instructions. In this case, we use KAPA HiFi (Roche).
4) Divide 100μL reaction into 4 PCR tubes containing 25μL reaction each.
5) Run gradient PCR (e.g. 60-70°C) to amplify the mCherry DNA sequence (711bp plus additional cloning adaptors).
6) Meanwhile, prepare 1.5% agarose gel for DNA electrophoresis. Prepare gel tray and combs for casting of gel. Add 0.75g of UltraPure agarose in 50mL of TAE buffer in a glass flask, microwave for 1-2min till fully dissolved. Next, add 5μL GelRed Nucleic Acid Stain (*see* Note 3).
7) Mix 2μL of each PCR reaction from step 5 with 1μL of NEB 6x gel loading dye. Load the samples into 4 wells of the agarose gel. Load 5μL of DNA ladder in an empty well and run DNA electrophoresis at constant 100V for 30min or until the DNA ladder is resolved.
8) Image agarose gel on a GelDoc system, repeat PCR if necessary with the appropriate annealing temperature.
9) Clean up the PCR reaction using a PCR cleanup kit (e.g., Macherey-Nagel NucleoSpin Gel and PCR Clean-up Kit) following the manufacturer’s instructions:
  - Add 2 volumes of Buffer NTI to 1 volume of sample i.e. 200μL of Buffer NTI to 100μL PCR products.
  - Transfer samples to a NucleoSpin Gel and PCR Clean-up Column in a 2mL collection tube.
  - Centrifuge for 30s at >10,000rcf. Discard flow-through and place column back into collection tube.
  - Add 700μL Buffer NT3 (prepared with 96-100% ethanol as indicated on the bottle) to the column.
  - Centrifuge for 30s at >10,000rcf. Discard flow-through and place column back into collection tube.
  - Repeat washing steps d and e.
  - Centrifuge for 30s at >10,000rcf to dry column, discard collection tube.
  - Place the spin column into a new 1.5mL microcentrifuge tube. Elute DNA with 30μL water and incubate at room temperature for 1min.
  - Centrifuge for 1min at >10,000rcf to elute DNA.
  - Measure concentration of eluted DNA and store at -20°C for future use.

### 3.3 Molecular Cloning

1) Prepare two restriction digest reaction mix for the PCR product (mCherry DNA fragment) and vector (e.g., pSO1070):
2) Perform restriction digest for 1h at the optimal temperature. This is 37°C for BamHI-HF and NotI-HF (*see* Note 4).
3) Add 6μL of NEB 6x gel loading dye and run the restriction digest reaction on a 1.5% agarose gel (*see* Subheading 3.2 steps 6-7). Excise the products from the gel using a clean scalpel. There should be one band at approximately 700bp for the PCR digest and two bands for the vector digest corresponding to GFP and the rest of the vector.
4) Extract and clean up the products using a PCR/Gel cleanup kit (e.g., Macherey-Nagel NucleoSpin Gel and PCR Clean-up Kit) following the manufacturer’s instructions (*see* Subheading 3.2 step 9). Dissolve the excised gel band in 2 volumes NTI buffer by heating in a 70°C heat block. Elute final product with 20-25μL of water.
5) Measure concentration of eluted DNA. Store at -20°C for future use.
6) Ligation of insert into vector *(*see* Note 5): Table 6: Ligation Reaction
7) Mix by gentle pipetting, incubate at room temperature for 1h.
8) Add ligation mix directly to a tube of thawed competent *E. coli* cells (e.g., NEB 5-alpha Competent *E. coli*).
9) Incubate on ice for 30min, heat shock at 42°C for 30s before returning tube to ice for 2min.
10) Recover in SOC media for 30min at 37°C.
11) Plate cells on LB plates containing 100μg/mL ampicillin and incubate overnight at 37°C.

### 3.4 Miniprep and analytical digestion

1) In 13mL snap-cap tubes, inoculate 4 colonies of transformed *E. coli* separately into 3mL LB media containing 100μg/mL ampicillin.
2) Grow bacteria cultures overnight at 37°C with constant shaking.
3) Pellet cells by centrifugation at >4000rcf for 5min at room temperature. Decant medium.
4) Use a miniprep kit of choice to purify plasmid DNA (e.g., Thermo Scientific GeneJet Plasmid Miniprep Kit) following the manufacturer’s instructions:
  - Add 250μL Resuspension Solution to the pelleted cells.
  - Resuspend cells by pipetting and transfer to a fresh microcentrifuge tube.
  - Add 250μL Lysis Solution and invert the tube 4-6 times to mix.
  - Add 350μL Neutralization Solution and invert the tube 4-6 times to mix.
  - Centrifuge tubes for 5min at >10,000rcf at room temperature.
  - Transfer the supernatants to separate GeneJet Spin Columns.
  - Centrifuge columns for 1min at >10,000rcf. Discard the flow-through.
  - Add 500μL of Wash Solution to the column.
  - Centrifuge columns for 1min at >10,000rcf.
  - Discard the flow-through.
  - Repeat steps h-j again.
  - Centrifuge the empty column for 1min at >10,000rcf to dry the column.
  - Transfer the column to a fresh microcentrifuge tube.
  - Add 50μL of PCR-grade water to the column and incubate for 2min.
  - Centrifuge for 2min at >10,000rcf to elute plasmid DNA.
  - Measure concentration of eluted DNA. Store at -20°C for future use.
5) Prepare a restriction digestion reaction master mix:
6) Aliquot 13μL of the master mix into 4 PCR or microcentrifuge tubes.
7) Add 2μL of plasmid DNA from the 4 minipreps from step 5 to each 13μL analytical digest reactions.
8) Incubate digestion reactions at optimal temperature for the restriction enzymes of choice for 30min (e.g. 37°C) (*see* Note 4).
9) Add 3μL of NEB 6x gel loading dye to each restriction digest, mix well.
10) Prepare 1.5% agarose gel for DNA electrophoresis (*see* Subheading 3.2 step 6).
11) Load all 18μL of the restriction digest from step 9 into 4 wells of the agarose gel. Load 5μL of DNA ladder and run DNA electrophoresis at constant 100V for 30min.
12) Image the agarose gel. Positive clones should have a band corresponding to the mCherry insert of approximately 700bp and a larger band corresponding to the rest of the vector.
13) Sanger sequence the positive clones to confirm the absence of unwanted mutations. Store the plasmids at -20°C for future use.

### 3.5 *S. pombe* chemical transformation

1) Linearize vector at the *ura4* marker with StuI restriction enzyme (see Subheading 3.3 Table 5). Purify the linearized plasmid using a PCR clean up kit (*see* Subheading 3.2 step 9) and measure the DNA concentration.
2) Grow 20mL of *S. pombe* strains of required genotypes (e.g., Nem1-mNeonGreen expressing cells) to OD_595_=0.5 or 1×10^7^ cells/mL, typically in Yeast Extract with Supplements (YES) medium.
3) Prepare reagents for chemical transformation. LiAc-TE (0.1M lithium acetate, 10mM Tris pH 7.5, 1 mM EDTA) and LiAc-TE-PEG (LiAc-TE plus 40% PEG4000).
4) Pellet cells via centrifugation at 1000rcf for 2min and remove supernatant.
5) Wash cells by resuspending pellet in 20mL of water followed by centrifugation at 1000rcf for 2min. Decant supernatant.
6) Resuspend cell pellet in 1mL water and transfer to a clean microcentrifuge tube.
7) Pellet cells via centrifugation at 1000rcf for 2min and remove supernatant.
8) Wash cells once in 1mL of LiAc-TE followed by centrifugation at 1000rcf for 2min. Remove supernatant by aspirating with a pipette.
9) Resuspend cell pellet in 100μL of LiAc-TE. Add 2μL of carrier DNA (10mg/mL Sonicated Salmon Sperm DNA) and 1-2μg linearized plasmid DNA to the cell mixture.
10) Add 260μL of LiAc-TE-PEG and mix gently.
11) Incubate for 30-60min at 30°C.
12) Add 43μL of DMSO and incubate cells at 42°C for 5min.
13) Pellet cells via centrifugation at 1000rcf for 2min and wash once with 1mL of water. Resuspend washed cells in 500μL of water and plate on two Edinburgh Minimum Media (EMM) lacking uracil (EMM ura^-^) selective plates. Incubate at 30°C for 3-4 days.
14) For a more detailed *S. pombe* transformation protocol, refer to this protocol by Moreno and colleagues [33]. Other transformation protocols, such as electroporation, can be used [34].

### 3.6 Selection and genotyping

1) *S. pombe* colonies will have formed following 3-4 days of growth on selective medium. We typically select 8 colonies and restreak them on a fresh EMM ura^-^plate using a sterile inoculation loop or pipette tip. Incubate plate in a 30°C incubator for 1-2 days.
2) When restreaked colonies have grown sufficiently, perform genomic DNA extraction as described below:
3) Scrape a single yeast colony of approximately 2mm in diameter from the agar plate.
4) Lyse cells and extract genomic DNA using a Genomic DNA extraction kit (e.g., MasterPure Yeast DNA Purification Kit, Biosearch Technologies) following the manufacturer’s instructions:
  - Add 300μL of Yeast Cell Lysis Solution to the microcentrifuge tube of yeast cells. Resuspend cells by pipetting up and down repeatedly.
  - Incubate the resuspended cells at 65°C for 15min.
  - Place the samples on ice for 5min.
  - Add 150μL of MCP Protein Precipitation Reagent and vortex for 10s.
  - Pellet cellular debris by centrifugation for 10min at >10,000rcf.
  - Transfer supernatant to a clean microcentrifuge tube and add 500μL of isopropanol. Mix thoroughly by inversion.
  - Pellet the DNA by centrifugation for 10min at >10,000rcf.
  - Decant the supernatant, wash the DNA pellet with 500μL of 70% ethanol.
  - Pellet the DNA by centrifugation for 5min at >10,000rcf.
  - Decant and remove any remaining ethanol by pipetting.
  - Resuspend the DNA in 50μL of PCR-grade water.
  - Measure DNA concentration.
5) Perform genotyping using primers flanking the integration site to confirm the integration of vector in the *ura4* locus.
6) Alternatively, strains containing the integrated lipid sensors can be crossed with strains containing mutants or markers of interest (*see* Moreno *et al*. [33])
7) Freeze positive clones for future use: scrape remaining restreaked yeast containing the integrated vector from the selective plate using a 10μL inoculation loop and transfer it into a cryotube containing 1mL of 50% glycerol and 50% YES medium. Resuspend yeast by pipetting up and down and store at -80°C for future use.

### 3.7 Quick imaging to check for the presence of the lipid sensor

**Figure 3:**
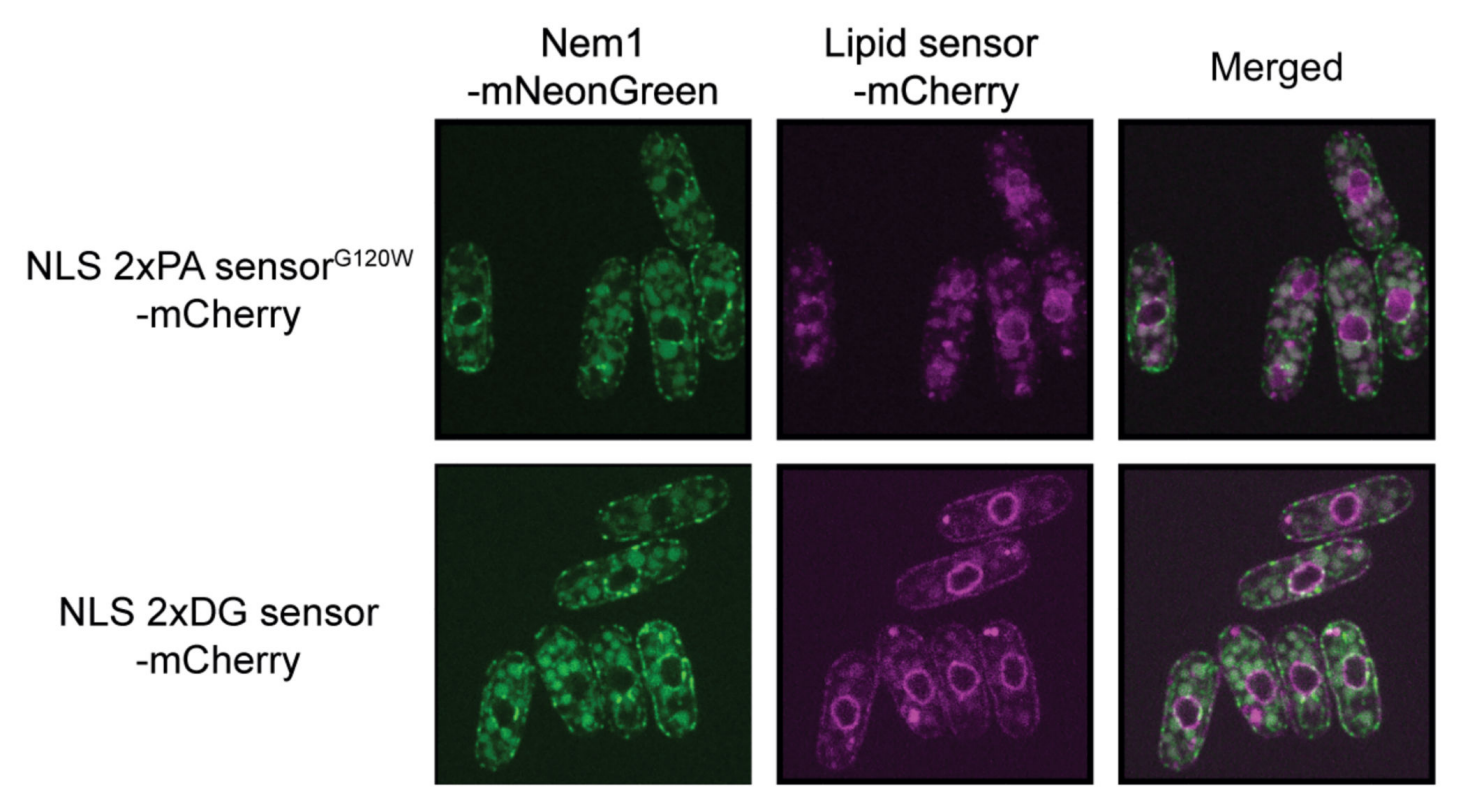
Single-plane spinning-disk confocal images of *S. pombe* cells co-expressing the ER-localized Lipin phosphatase Nem1-mNeonGreen together with the mCherry tagged NLS PA and DG sensors. Scale bars: 5 µm.

1) When performing a quick screen for the presence of the lipid sensor of interest, restreaked yeast colonies can be checked under a fluorescence microscope using the appropriate imaging settings for the genetically encoded fluorophore of choice, as follows:
2) Pipette 2μL of YES or EMM medium onto a glass microscope slide.
3) Using a pipette tip, scrape a small amount of restreaked yeast from the selective plates and dab it onto the medium droplet 3-4 times to resuspend it. Place a glass cover slip on the droplet and check for fluorescence.
4) An example of *S. pombe* expressing a protein of interest, the lipin phosphatase Nem1, tagged with mNeonGreen together with the mCherry-tagged PA or DG lipid sensor is shown in Figure 3.

### 3.8 Live cell imaging

1) Frozen yeast stocks can be restreaked directly on YES plates or EMM ura^-^ plates by scraping a small amount of frozen material with a clean pipette tip. Incubate at 30°C for 1-3 days until colonies are formed. We typically restreak these once again allowing them to grow overnight. This step ensures that cells are outside of stationary phase when inoculated in liquid media for imaging.
2) Inoculate freshly restreaked cells in 10-20mL appropriate liquid medium. Culture in a shaking incubator overnight at 30°C. Use cells for imaging at OD_595_=0.3-0.6 or ~1×10^7^ cells/mL. If cell culture overgrew the target OD_595_, dilute it to OD_595_=0.1-0.2 and continue growing until the required density is reached.
3) Centrifuge 1 ml of cell culture in a microcentrifuge tube at 1000rcf for 2min and remove supernatant by decanting (*see* Note 6).
4) For snapshots, resuspend the pellet in 20-50μL of media and place 2μL of cells on a glass microscope slide, cover with a cover slip and image cells, as appropriate.
5) For imaging *S. pombe* cells over a longer periods of time to produce time-lapse movies or montages (see Figure 3 in [4]), or for quantification of changes in lipid sensor distribution over time, live cell imaging on agarose pads should be performed. This is described in detail by Pemberton [35] but we provide a brief protocol below.
6) Prepare 20mL YES media with 1% agarose. Dissolve the agarose by briefly microwaving the solution. Aliquot 1mL of the solution into microcentrifuge tubes for future use.
7) To prepare agarose pads for imaging, melt an aliquot from step 6 using a 95°C heat block for 5-10min. If further use of molten agarose medium is required, transfer the aliquot to a 65°C heat block to prevent it from burning (*see* Note 7).
8) Meanwhile, prepare a glass slide and two pieces of cardboard or tape to act as spacers. Place the spacers on the two ends of the glass slide.
9) Mix the melted aliquot of 1% agarose by inverting the tube, and pipette 50-80μL onto the middle of the glass slide.
10) Gently place another glass slide on the top, flattening the agarose drop. The second slide should rest on the two spacers on either side (*see* Note 8).
11) After about 30-60s, the agarose pad will have solidified. Gently slide the second glass slide off, keeping the agarose pad in the middle of the first slide.
12) Allow the agarose pad to dry for 1min. Prepare *S. pombe* cells for imaging as outlined above (steps 1-3). Gently resuspend the cell pellet in 20-50μL of media and place 0.8-1μL of cells on the agarose pad.
13) Gently cover the pad with a glass coverslip, avoiding air bubbles, and seal it with wax or nail polish.
14) Incubate the prepared slide in the temperature-controlled microscope set-up (e.g., using an Okolab Cage Incubator) to allow it to acclimatize to the imaging temperature (*see* Note 9).
15) Image cells as required.

### 3.9 Optional Spectral Autofluorescence Image Correction By Regression (SAIBR) protocol

1) To reduce autofluorescence in the green channel we recommend exploring the SAIBR method [36]. The SAIBR plugin for FIJI, together with detailed instructions and sample datasets is available online at https://github.com/goehringlab/saibr_fiji_plugin
2) Briefly, image the strains containing GFP-tagged lipid sensors using the green channel using appropriate microscopy settings. At the same time, image the strains in the red channel with the same settings for autofluorescence correction in SAIBR. Additionally, image 3 to 5 fluorophore-negative samples (e.g., wild-type cells) using the green and red channels with the same settings for calibration of the plug-in.
3) Download and install the plug-in from the link above
4) Open the plug-in in *Plugins -> SAIBR*
5) Calibrate using 3 to 5 fluorophore-negative control samples (e.g., wild-type cells). Set the green channel as the Primary Channel and red channel as the Predictor Channel.
6) Specify regions of interest (ROI) around the cells.
7) Run calibration.
8) Check the scatterplot for a good 1:1 relationship across the range of values for all images, and a high R^2^ value of >0.9
9) Adjust the Gaussian Blur value and rerun the calibration if necessary.
10) Once satisfied with the calibration, save calibration and return to the main menu.
11) Open the image files to be corrected.
12) Check that the parameters for correction are set according to the calibration performed. Set the green channel as the Primary Channel and the red channel as the Predictor Channel.
13) Click on *Run correction* to perform autofluorescence correction on the image.
14) Repeat the process for the other image files to be processed.

### 3.10 Quantification of PA and DG sensor intensity at the INM

1) We typically quantify PA and DG sensor intensities at the INM (or other cellular locations, such as the cortex) using the segmented line function in FiJI/ ImageJ [37]:
2) Right click on the Straight Line function button to switch to the Segmented Line function. Double click the Segmented Line function button to adjust the line width to 5 pixels or an appropriate size to encompass the region of interest.
3) Use the Set Measurements function under the Analyze menu and set it to display the mean grey value, perimeter and area, and any other measurements of interest.
4) Select a cell of interest (e.g., a dividing cell)
5) Outline the region of interest (i.e., the fluorescence from the lipid sensor at the INM), and use the Measure function (under the Analyze menu). The Measure function will display the values as selected in step 3.
6) Repeat this measurement for every time point in the montage taken for the cell of interest.
7) Transfer the data to a data analysis software of choice (e.g., Microsoft Excel, GraphPad Prism).
8) The sensor intensity at the INM can be divided by the intensity at the cortex to normalize the readings, to account for photobleaching.
9) Next, plot the rate of sensor signal decay at the INM against time for the cell of interest.
10) Repeat steps 4 to 9 for other dividing cells.
11) Linear regression of the sensor signal decay of each individual cell can be calculated. The gradient calculated is the rate of sensor intensity decay at the INM.

## Figures and Tables

**Table 1:** Plasmid information

Plasmid	Insert	Description
pSO1074	PA sensor	pJK210- promoter^*cdtc15*^-PA sensor (Opi1^111-189,^ ^G120W^ 2xAH)-GST-GFP
pSO1070	NLS PA sensor	pJK210- promoter^*cdtc15*^-NLS PA sensor (NLS^SV40^ - Opi1^111-189, G120W^ 2xAH-NLS^SV40^)-GST-GFP
pSO1079	DG sensor	pJK210- promoter^*cdtc15*^-DG sensor (PKCβ^31-158^ 2xbinding domain)-GST-GFP
pSO1080	NLS DG sensor	pJK210- promoter^*cdtc15*^-NLS DG sensor (NLS^SV40^ - PKCβ^31-158^ 2xbinding domain- NLS^SV40^)-GST-GFP

**Table 2:** Yeast strain information

Strain	Lipid Sensor	Genotype
SO8772	PA sensor	*ura4+::*promoter^*cdtc15*^-PA sensor-GST-GFP *ade6-704 ura4-294 leu1-32 h-*
SO8760	NLS PA sensor	*ura4+::*promoter^*cdc15*^-NLS PA sensor-GST-GFP *ade6-704 ura4-294 leu1-32 h-*
SO8766	DG sensor	*ura4+::*promoter^*cdc15*^-DG sensor-GST-GFP *ade6-704 ura4-294 leu1-32 h-*
SO8767	NLS DG sensor	*ura4+::*promoter^*cdc15*^-NLS DG sensor-GST-GFP *ade6-704 ura4-294 leu1-32 h-*

**Table 3:** KAPA HiFi PCR Reaction

Reagent	Volume
5x KAPA HiFi Buffer	20μL
10mM dNTP mix	3μL
10μM Forward Primer	3μL
10μM Reverse Primer	3μL
Template DNA (e.g., plasmid with mCherry sequence)	***1-5ng***
1U/μL KAPA HiFi DNA polymerase	2μL
PCR-grade water	Up to 100μL
**Total Volume**	**100μL**

**Table 4:** KAPA HiFi PCR cycling protocol

Step	Temperature	Duration	Cycles
Initial denaturation	95°C	3min	1
Denaturation	98°C	20s	34
Annealing	60-70°C	15s	
Extension	72°C	15s/kb	
Final extension	72°C	5min	1
Completion	12°C	∞	1

**Table 5:** Restriction Digest Reaction

Reagent	Volume
CutSmart Buffer	3μL
DNA	***1-2μg***
Restriction Enzyme #1 (BamHI-HF)	1μL
Restriction Enzyme #2 (Notl-HF)	1μL
PCR-grade water	Up to 25μL
**Total Volume**	**30μL**

**Table 6:** Ligation Reaction

Reagent	Volume
T4 DNA ligase buffer (10x)	2μL
Insert DNA	13μL*
Vector DNA	4μL*
T4 DNA ligase	1μL
**Total Volume**	**20μL**

**Table 7:** Analytical Digest Master Mix

Reagent	Volume
CutSmart Buffer	6 μL
H_2_O	42μL
Restriction Enzyme #1 (BamHI-HF)	2μL
Restriction Enzyme #2 (Notl-HF)	2μL
**Total Volume**	**52μL**

## 4 Notes

1 Care should be taken when choosing an appropriate promoter for overexpression of PA and DG sensors, as strong overexpression, e.g., using the strong *tdh1* promoter [38] may lead to NE morphology defects (see Figure S2D in [4]).

2 It should be noted that further optimization of the lipid sensors might be necessary for different species. For example, we have observed that the abundance of both nuclear and cytoplasmic PA sensors is significantly weaker in *S. japonicus*, as compared to *S. pombe* [4]. This is not due to the promoter usage, as the DG sensors driven by the same regulatory elements are expressed well. It is possible that the sensor stability could be affected by differences in acyl chain composition of membrane lipids in the two species [4, 39].

3 Volume of agarose gel depends on the DNA gel electrophoresis system used.

4 If the restriction enzymes have two different optimal temperatures, perform the digest for 30min at the lower temperature followed by 30min at the higher temperature.

5 Ligation ratio 1:3 up to 1:8 of vector:insert. Use an online ligation calculator (e.g., the NEBioCalculator at https://nebiocalculator.neb.com/#!/ligation) to determine the amount and volume required, as this depends on both the concentration and size of the respective fragments.

6 Avoid centrifuging cells for imaging at high speeds to reduce stress.

7 Once molten, the agarose-containing medium kept at 65°C can be used to make more agarose pads on the same day.

8 Optimal agarose pad thickness is approximately 1-2mm. It can adjusted by using spacers of appropriate thickness.

9 Live cell imaging is typically performed at 30°C for *S. pombe* cells, unless working with temperature-sensitive mutants or temperature shift-based experiments.
